# Effect of Mineral Powders on the Properties of Foam Concrete Prepared by Cationic and Anionic Surfactants as Foaming Agents

**DOI:** 10.3390/ma17030606

**Published:** 2024-01-26

**Authors:** Qi Liu, Huanghua Chen, Shiyu Fang, Jin Luo

**Affiliations:** State Key Laboratory of Featured Metal Materials and Life-Cycle Safety for Composite Structures, School of Civil Engineering and Architecture, Guangxi University, Nanning 530004, China; chenhuanghua@st.gxu.edu.cn (H.C.); 2210391020@st.gxu.edu.cn (S.F.); 2210391076@st.gxu.edu.cn (J.L.)

**Keywords:** foam concrete, mineral powders, pore characteristics, thermal conductivity, FTIR

## Abstract

Foam concrete is a type of cement mortar in which air bubbles are introduced using an appropriate foaming agent. The complex conditions for the preparation of solid particle stabilized foams limit their wide application in construction. In this study, a method of adding small amounts of calcite (Cal) and muscovite (Mus) to the cement paste matrix is proposed to improve the properties of foam concrete prepared with cationic and anionic surfactants as foaming agents. The effects of mineral powders on the flowability, compressive strength, water absorption, pore characteristics, thermal conductivity and frost resistance of foam concrete were investigated and the enhancement mechanism was revealed by the results of XRD, low-field nuclear magnetic resonance (LF-NMR), Fourier transform infrared spectroscopy (FTIR) and SEM. The results showed that the mineral powders interacted with anionic and cationic surfactants through physical adsorption. Whether anionic or cationic surfactants were used as foaming agents, the addition of mineral powders promoted the formation of shell-like structures around the foam, thus enhancing the performance of foam concrete. As a result, the fluidity, compressive strength and frost resistance of the foam concrete increased, the water absorption and thermal conductivity decreased, and the average size of the pores decreased.

## 1. Introduction

Foam concrete is a kind of lightweight porous insulation material which has the advantages of low density, good workability, low thermal conductivity and good fire resistance. It has been widely used in construction. The properties of foam concrete are affected by its unique pore structure, which heavily depends on the foam properties [1]. Foam is an unstable system composed of a large number of bubbles, and its deterioration includes the discharge of liquid, the rupture of liquid film and Oswald ripening [2]. The instability of the foam can lead to the collapse of foam concrete and adversely affect the application of foam concrete in practical engineering [3,4,5,6,7].

Therefore, solid particles are used to prepare three-phase foam [8,9,10]. Three-phase foam has better stability than two-phase foam [11,12]. The main mechanism of particle-stabilized foam is that particles gather at the gas-liquid interface and decrease the contact area between bubbles. The dense particle film formed can inhibit the coalescence and disproportionation of the bubble [13]. Many studies have been carried out on the effect of particles as a stabilizer on foam stability [14].

However, the specific properties of three-phase foam are very dependent on particle sizes, concentrations, wetting properties, surfactant properties, and types [15,16,17]. Three-phase foam has been a research hotspot in the field of foam concrete in recent years. She et al. [18] showed that nano-silica (NS) and hydroxypropyl methylcellulose (HPMC) can prevent gas transfer and hinder physical drainage between the gaseous and liquid phases. Moreover, they found that the foam concretes prepared by incorporating nanoparticle-stabilized foams possess higher compressive strengths and narrower pore size distributions compared to normal foam concrete. Guo et al. [19] reported that a novel ultra-stable aqueous foam with a controllable structure and plateau borders was synthesized by adding polyvinyl alcohol (PVA) and nano-alumina (NA) complexes to the anionic surfactant sodium dodecyl sulfate (SDS). The interaction between SDS and nano-alumina (NA) improved the properties of foam concrete. Li et al. [20] investigated the waste limestone powder (WLP) processed by the wet grinding process (i.e., WGWLP, D50 = 281 nm), and wet-ground waste limestone powder (WGWLP) was used as a foam stabilizer to prepare high-stability foams for the preparation of high-stability and high-strength foam concrete. Krämer et al. [21,22,23] demonstrated that three-phase foam materials integrating NA, nano-silica (NS) and carbon nanotubes somewhat improved the properties of foam concrete.

The high dosage of nanoparticles and their complicated preparation limited the applicability of this material. Although the foams prepared in the above-described studies had good stability, there remains a problem with applying the technology in practical engineering. The air compressors are often used to prepare foams. However, this means that the method of adding solid particles to the foaming agent is inconvenient. Therefore, it is necessary to solve this problem from other angles. Therefore, it was feasible to modify foam concrete by adding minerals to the cement paste matrix. Thus, foam can be reinforced by mineral powders after the foam concrete is cast.

In this paper, two kinds of mineral powders, calcite and muscovite, were mixed with cement, respectively, to prepare the cement paste. The foam was prepared by SDS (anionic surfactant) or DTAB (cationic surfactant) and then added to the cement paste to prepare foam concrete. The properties of foam concrete were studied, such as compressive strength, water absorption, pore characteristics, thermal conductivity and frost resistance. The hydration and micropore of the specimens were also analyzed using X-ray diffraction and low−field NMR. FTIR was used to check the adsorption state of mineral powders onto cationic and anionic surfactants. Overall, this investigation is aimed at certifying how mineral powders enhance the properties of foam concrete.

## 2. Materials and Methods

### 2.1. Materials

**Figure 1:**
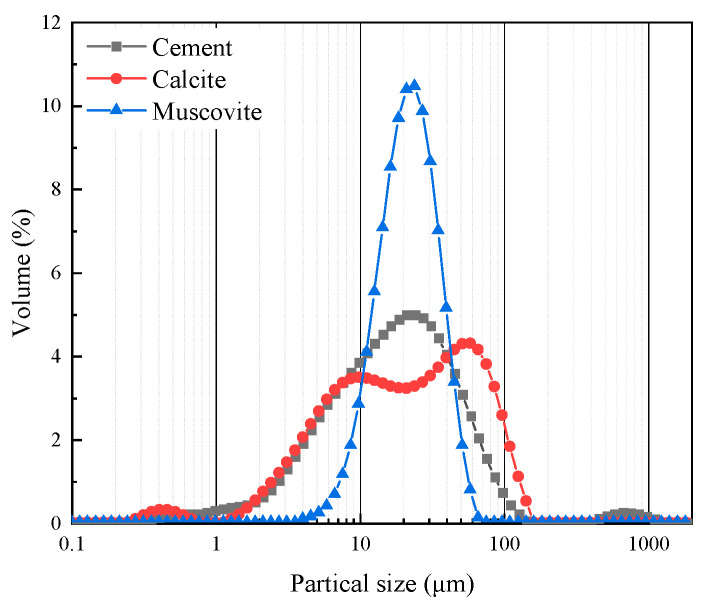
Particle size distributions of raw materials.

**Figure 2:**
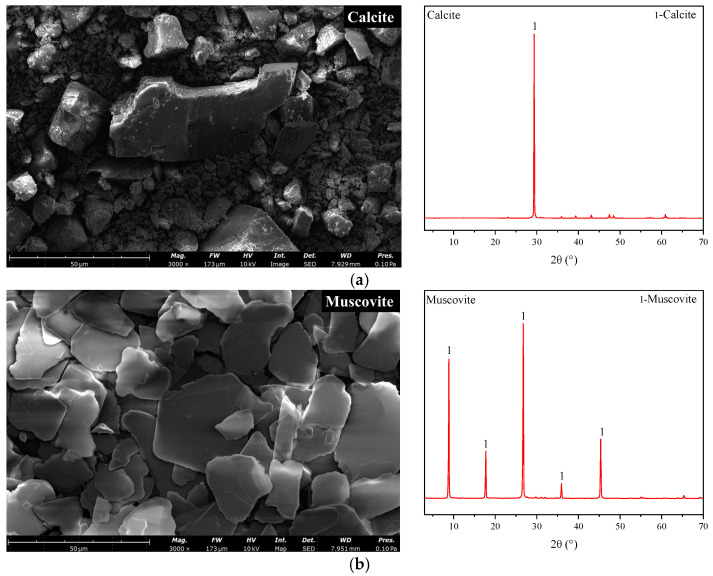
XRD patterns and (3000×) SEM image of (**a**) calcite and (**b**) muscovite.

Ordinary Portland cement P.O 42.5 was provided by Anhui Conch Cement Co., Ltd. (Wuhu, China). The basic properties of cement are listed in Table 1. Calcite (Cal) and muscovite (Mus) used in the study were obtained from Shanlinshiyu Mineral Products Co., Ltd. (Guzhang, China). The particle size distributions of the raw materials are shown in Figure 1. X-ray diffraction (XRD) patterns and SEM images of calcite and muscovite are shown in Figure 2.

**Figure 3:**
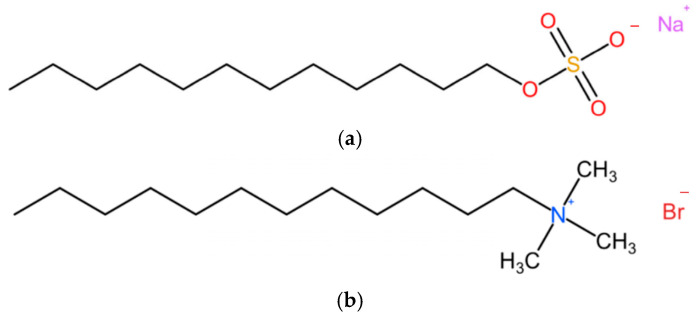
Molecular structures of the two foaming agents: (**a**) Sodium dodecyl sulfate (SDS), anionic surfactant and (**b**) Dodecyl trimethyl ammonium bromide (DTAB), cationic surfactant.

Two representative surfactants with alkyl tails of the same length were used as foaming agents and provided by Shanghai Macklin Biochemical Technology Co., Ltd. (Shanghai, China). Sodium dodecyl sulfate (SDS) was selected as an anionic surfactant. Dodecyl trimethyl ammonium bromide (DTAB) was selected as a cationic surfactant and the molecular structures are shown in Figure 3. The water used in the experiment was tap water. The concentration of foaming agents was controlled at 10 g/L to decrease concentration interference [17].

**Figure 4:**
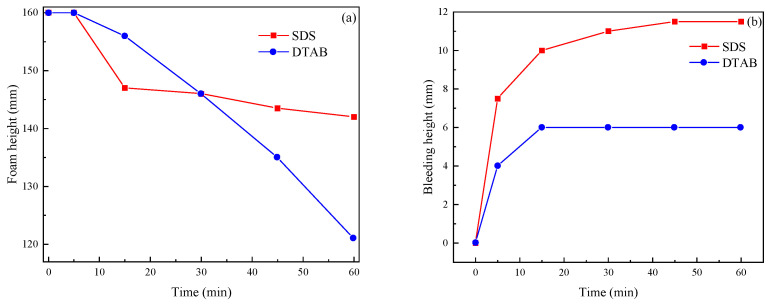
(**a**) Foam height and (**b**) bleeding height of the SDS 10 g/L and DTAB 10 g/L.

The two foaming agents were foamed using the F80 portable bubbler. The foam densities of DTAB and SDS are 22.38 kg/m^3^ and 37.77 kg/m^3^. The changes in settling distance and water secretion of prefabricated foam within 1 h were measured according to Chinese standard JC/T2199-2013 [24] Foaming agents for foamed concrete, China, 2013, and the results are shown in Figure 4. The foam heights of the SDS and DTAB were 142 mm and 121 mm and the bleeding heights were 11.5 mm and 6 mm, respectively.

### 2.2. Preparation

The mixture proportion of foam concrete is calculated according to the target density. The design method (of 1 m^3^ foam concrete) used in the study is described as follows [25]:

(1) $$\rho_{d}=S_{a}m_{c}$$

(2) $$V_{2}=K\left(V-V_{1}\right)=K\left[V-\left(\frac{m_{c}}{\rho_{c}}+\frac{m_{w}}{\rho_{w}}\right)\right]$$

where $\rho_{d}$(kg/m^3^) is the target density of the foam concrete; $S_{a}$ is the empirical coefficient, which is 1.2 for standard 42.5 Portland cement. $m_{c}$(kg) and $m_{w}$(kg) are the masses of cement and water separately. *V*(m^3^) is the volume of foam concrete, equal to 1 m^3^. $V_{1}$(m^3^) and $V_{2}$(m^3^) are the volumes of cement paste and foam, respectively. $\rho_{c}$(kg/m^3^) and $\rho_{w}$(kg/m^3^) are the densities of cement and water. In this paper, $m_{w}$ = 0.5 $m_{c}$. *K* is a coefficient decided by the foam quality, equal to 1.60 in this study [26].

Foam concrete with two types of foaming agents was cast, and the target density of the foam concrete was 800 kg/m^3^. The mixture proportions and actual density of foam concrete are given in Table 2.

**Figure 5:**
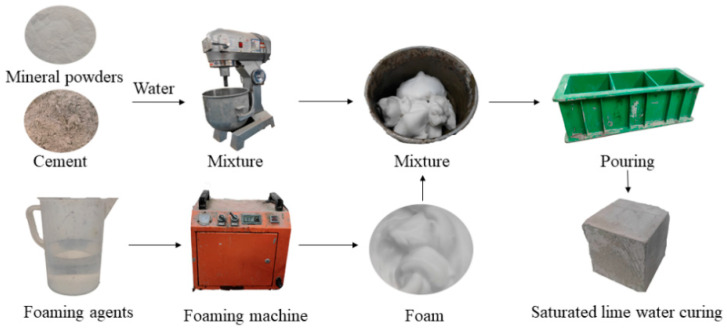
Process of the preparation of foam concrete.

At first, according to the mix proportions, cement paste with a water cement ratio of 0.5 was prepared. Meanwhile, the two kinds of foam were produced by a foaming machine and were added to the cement paste and mixed for 1 min by a mixer at about 180 r/min. Then, the paste was poured into molds with a size of 100 mm × 100 mm × 100 mm. The specimens were demolded at 1 day and cured with a temperature of 20 ± 3 °C and a relative humidity of 90% until test time. The process is shown in Figure 5.

### 2.3. Methods

The fluidity of the fresh foam concrete, compressive strength, dry density and water absorption capacity of hardened foam concrete were measured according to Chinese standard JGJ/T 341-2014 [27], Technical specification for application of foamed concrete, China, 2014 Three specimens were tested for each mix, and the average of three measurements with the standard deviation was recorded.

**Figure 6:**
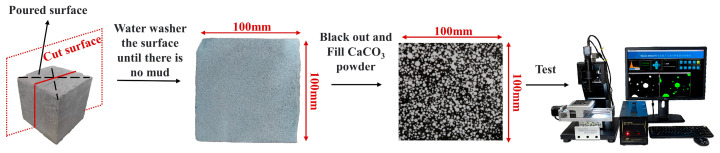
Sampling flow chart of pore characteristics.

For the pore characteristics, the sampling process was adopted from Ref [28], as is shown in Figure 6.

The thermal conductivity test was carried out according to Chinese standard GB/T 10294-2008 [29], using a DRM-2 thermal conductivity measuring instrument.

The Frost resistance was tested according to the JGJ/T 341-2014 [27] with some modifications. The specimen size is 100 mm × 100 mm × 100 mm, with three specimens in each group. After 28 days of standard maintenance, the specimens were placed in a drying box at a temperature of (60 ± 5) °C and dried to constant weight. After the specimen blocks were cooled to room temperature, they were completely submerged and immersed in a sink, saturated with water for 48 h, and then placed in a refrigerator at a temperature of −20 °C. The time used for freezing was 7 h and the time used for thawing was 6 h. After 30 freeze–thaw cycles were completed, the test blocks were transferred to a desiccator at a temperature of (60 ± 5) °C and dried to constant weight. Then, the compressive strength test was carried out.

XRD analysis of specimens was carried out using a Miniflex600 X-ray diffractometer (Rigaku, Tokyo, Japan) to study the influence of mineral powder on the hydration products of foam concrete. The diffractometer used a copper target, a tube voltage of 40 kV, and a tube current of 40 mA. The scanning angle range was 5° to 65° 2θ and the scanning step size was 0.01 2θ. The total scanning time was 30 min.

The FTIR test was carried out using an IRTracer-100 Fourier Transform Infrared spectrometer (SHIMADZU, Kyoto, Japan) to check the adsorption state of mineral powders onto cationic and anionic surfactants, with a wave number range of 400–4000 cm^−1^ and a resolution of 2 cm^−1^. The processing method of the mineral specimens to be tested was as follows: 3 g of single mineral was dispersed in 100 mL of a model pore solution of cement pastes consisting of 20 mM Ca (OH)_2_ and 150 mM K_2_SO_4_ [30]. Then, 6 g of anionic and cationic surfactants were added. After the pulp had been thoroughly stirred for 3 h on a magnetic stirrer, it was filtered and repeatedly washed with deionized water. Finally, the mineral specimens were dried in a constant temperature vacuum drying oven at 105 °C for 6 h.

The LF-NMR test was performed using a MacroMR12-110H-1 instrument (NIUMAG, Suzhou, China) to qualitatively characterize the pore structure distribution in the foam concrete with a constant magnetic field of 0.49 T, a 45 mm coil and an operating frequency of 20 MHz. The specimens were vacuum-filled with water 1 day before the test, and the *T*_2_ relaxation times of water in the saturated specimens were analyzed to characterize the distribution of pores [31].

(3) $$\frac{1}{T_{2S}}=\rho_{2}\frac{S}{V}$$

(4) $$\frac{1}{T_{2}}\approx \frac{1}{T_{2s}}=\rho_{2}\frac{S}{V}$$

where $T_{2S}$ represents the surface relaxation contribution of the specimens’ pores; $\rho_{2}$ represents the surface relaxation strength of the fluid and allows a qualitative analysis of the pore structure distribution.

## 3. Results

### 3.1. Cement Paste without Foam Properties

**Figure 7:**
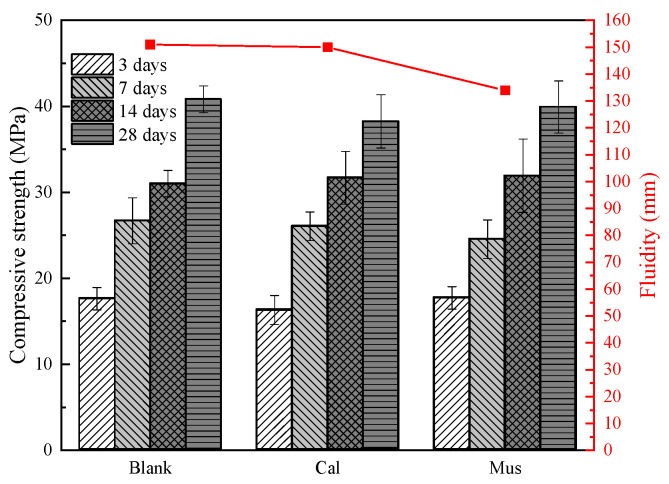
Compressive strength and fluidity of cement paste without foam.

We first tried to find out whether the properties of the cement paste matrix without foam are changed by mineral powders, and the results are shown in Figure 7. The addition of muscovite reduced the cement paste fluidity by 11% compared to the blank group. Muscovite is a layered silicate mineral with water absorption capacity [32]. Additionally, muscovite has a smaller particle size than cement particles. The larger specific surface area of muscovite increased the consumption of free water. Thus, the viscosity of the cement paste increased and there was a reduction in fluidity.

Furthermore, the compressive strength of cement paste increased gradually over time. The compressive strength of the blank group was 40.83 MPa at 28 days. With the addition of calcite and muscovite, the compressive strengths were 38.26 MPa and 39.94 MPa, respectively. This represents a reduction of 6.3% and 2.2% compared to the blank group. Overall, the mineral powders had little to no effect on the compressive strength. Additionally, the addition of muscovite decreased the fluidity of the cement paste.

### 3.2. Workability and Mechanical Properties

#### 3.2.1. Fluidity

**Figure 8:**
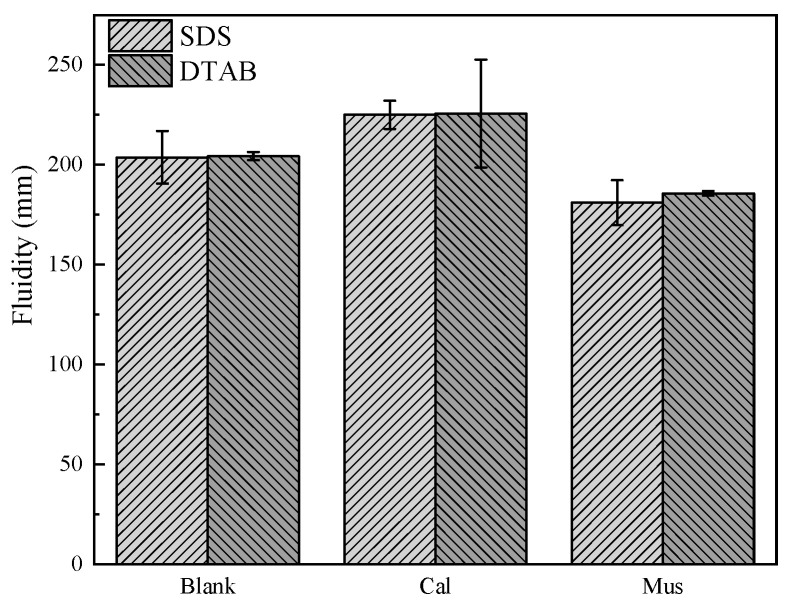
The effect of different mineral powders’ fluidity on foam concrete.

The effect of mineral powders on the fluidity of fresh foam concrete is shown in Figure 8. For the SDS group, the fluidity of S was 204 mm. S-Cal and S-Mus were 225 mm and 181 mm, respectively. S-Cal increased by 10.29% and S-Mus decreased by 11.27%. For the DTAB group, the fluidity of D was 204 mm. D-Cal and D-Mus were 226 mm and 186 mm, respectively. D-Cal increased by 10.78% and D-Mus decreased by 8.82%. Calcite particles are slightly larger than cement particles and are inert. Adding Cal reduces the free water required for the cement paste, increasing the flowability of fresh foam concrete. Section 3.1 concludes that the fluidity of cement slurry decreases with the addition of muscovite, resulting in the decreased fluidity of fresh foam concrete. Furthermore, the fluidity of fresh foam concrete did not differ much between the two foaming agents.

#### 3.2.2. Compressive Strength and Dry Density

**Figure 9:**
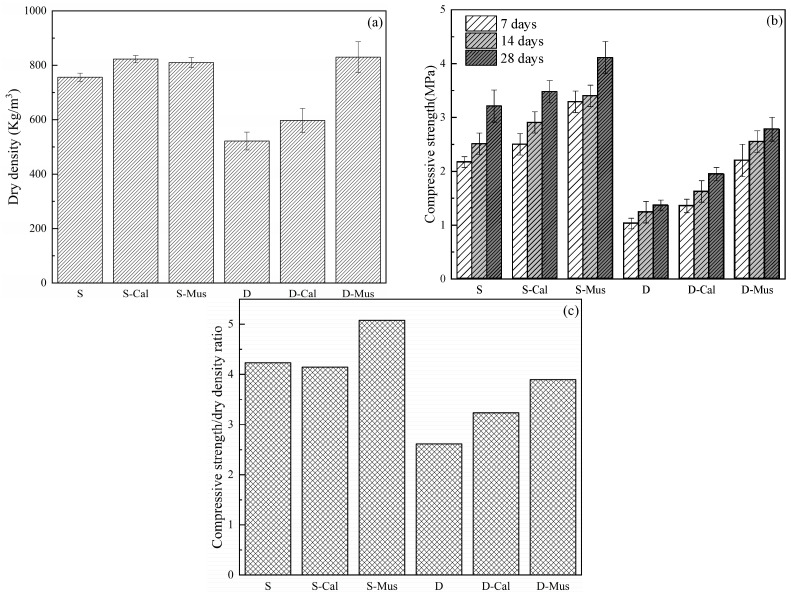
The effect of different mineral powders on the (**a**) dry density, (**b**) compressive strength and (**c**) compressive strength/dry density ratio of foam concrete.

The results of the effect of different mineral powders on the compressive strength, dry density and compressive strength/dry density ratio of foam concrete are shown in Figure 9. From Figure 9a, for the SDS group, the dry density of S was 758 kg/m^3^. S-Cal and S-Mus were 822 kg/m^3^ and 810 kg/m^3^, which increased by 8.44% and 6.86%, respectively. For the DTAB group, the dry density of D was 521 kg/m^3^. D-Cal and D-Mus were 597 kg/m^3^ and 830 kg/m^3^, which increased by 14.59% and 59.31%, respectively. The reason is that cement paste with added minerals powders will lead to water loss of the foam liquid film when mixing with the foam. The water absorption capacity of the mineral powders results in the reduction of the foam content in the fresh foam concrete, which in turn affects the dry density.

From Figure 9b, the compressive strength increased gradually over time. Meanwhile, the compressive strength of foam concrete increased with the addition of mineral powder. The addition of mineral powders caused a change in the dry density of specimens. To prevent the effect of change in dry density on the comparison of compressive strength, the effect of mineral powders on the compressive strength/dry density ratio was researched. For the SDS group, the compressive strength/dry density ratio of S was 4.23, and of S-Cal and S-Mus was 4.15 and 5.08, respectively. There was an increase of 20.94% in S-Mus. For the DTAB group, D was 2.61, and D-Cal and D-Mus were 3.32 and 3.37, respectively. There was an increase of 27.20% and 29.12%, respectively. The reason for the increase in strength is explained in Section 3.6.4.

Furthermore, the dry density of the samples in the DTAB group was lower than that in the SDS group due to being less dense and having higher-air-volume foam produced by DTAB as a foaming agent. Consequently, the specimens of the DTAB group had a higher porosity and lower dry density. Density is one of the major key factors that contribute to the compressive strength of foam concrete [33]. Thus, the compressive strength of specimens in the DTAB group is lower than that in the SDS group.

### 3.3. Physical Properties

#### 3.3.1. Water Absorption

**Figure 10:**
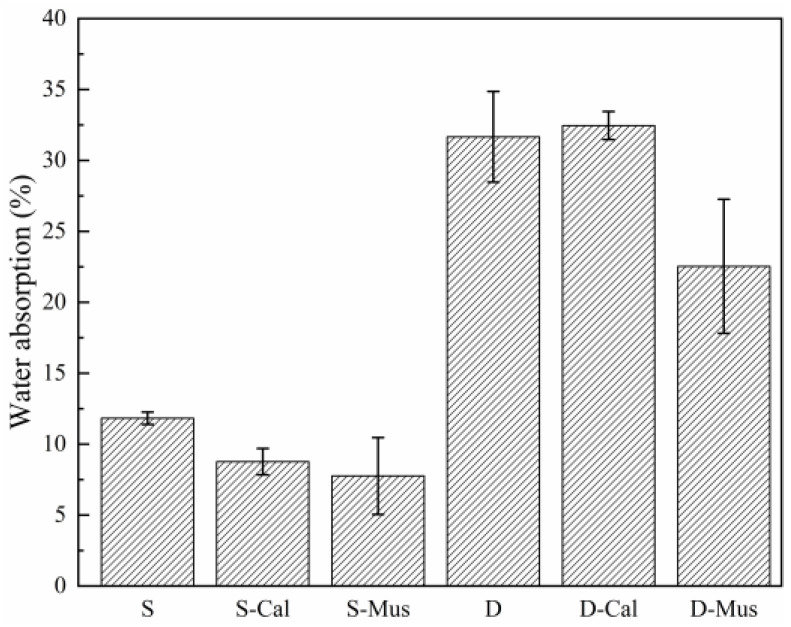
The effect of different mineral powders on the water absorption of foam concrete.

The results of the effect of different mineral powders on water absorption are shown in Figure 10. For the SDS group, the water absorption of S was 11.83%. S-Cal and S-Mus were 8.77% and 7.76%, which increased by 25.87% and 34.40%, respectively. For the DTAB group, the water absorption of D was 31.67%. D-Cal and D-Mus were 32.46% and 22.54%. There was a decrease of 28.83% in D-Mus. The water absorption in foam concrete is closely related to the presence of voids and pores [34]. The conclusion that particles can be used to fill the capillary pores of foam concrete and thus reduce water absorption was also obtained in the study by Song et al. [35]. Thus, the addition of mineral powders refines the pore size of the foam concrete and decreases water absorption. This is explained in Section 3.6.2. In addition, the water absorption in the DTAB group was higher than that in the SDS group, which may be explained by the fact that the specimens in the DTAB group had a loose stomatal wall structure. Thus, there are more capillary pores in the cement matrix. This is explained in Section 3.6.4.

#### 3.3.2. Pore Characteristics

The pore parameters of the specimens with SDS and DTAB groups are shown in Table 3 and Table 4. From Table 3, the number of pores is 4668 for S and 6601 and 5636 for S-Cal and S-Mus, which increased by 41.41% and 20.74%, respectively. For the percentage of pores measuring smaller than 500 μm, it was 71.4% for S and 74.9% and 76.4% for S-Cal and S-Mus, which increased by 4.90% and 7.00%, respectively. The porosity values of S were 35.93%, 42.46% and 38.71% for S-Cal and S-Mus, which increased by 18.17% and 4.74%. Due to the low porosity of the foam concrete prepared by SDS as foaming agent, it is sensitive to changes in the number of pores. Therefore, the addition of mineral powder increased the porosity. In addition, the average pore diameter was 35.93% for S and 42.46% and 38.71% for S-Cal and S-Mus, which decreased by 16.43% and 10.75%.

The reason is that when the prepared foam is mixed with the cement paste to which the mineral powder is added, the mineral powders absorb the free water of the foam liquid film due to its water absorption capacity, destroying part of the foam. The destroyed foam contains surfactant, which introduces new bubbles during fan blade mixing. Due to the adsorption between the mineral powders and the surfactant, the new bubbles introduced have mineral particles adsorbed on the surface, which in turn can increase the strength of the bubble film and prevent merging between the bubbles. At the same time, the size of bubbles introduced by stirring is approximately 500 μm or less. Therefore, the addition of mineral powder increased the number of air bubbles in the range of 500 μm or less.

From Table 4, the number of pores is 2770 for D and 3477 and 3692 for D-Cal and D-Mus, which is increased by 25.52% and 33.29%, respectively. For the percentage of pores measuring smaller than 500 μm, it was 52.1% for D and 48.1% and 49.8% for D-Cal and D-Mus, which increased by 7.68% and 4.41%, respectively. The porosity was 52.32% for D and 59.43% and 52.25% for D-Cal and D-Mus. There was an increase of 13.35% in D-Cal. In addition, the average pore diameter is 1.209 μm for D and 1.094 μm and 0.906 μm for D-Cal and D-Mus, which decreased by 9.51% and 25.06%. Due to the large porosity of the foam concrete prepared by DTAB as foaming agent, it is sensitive to the changes in the pores.

In summary, it can be concluded that the number of pores in the range of <1000 μm was increased with the addition of mineral powders, and thus the porosity was increased and the average pore size was decreased. Furthermore, the SDS group had lower porosity and average pore size compared to the DTAB group, which explains the dry density of the samples in the SDS group being more than in the DTAB group.

### 3.4. Thermal Conductivity

**Figure 11:**
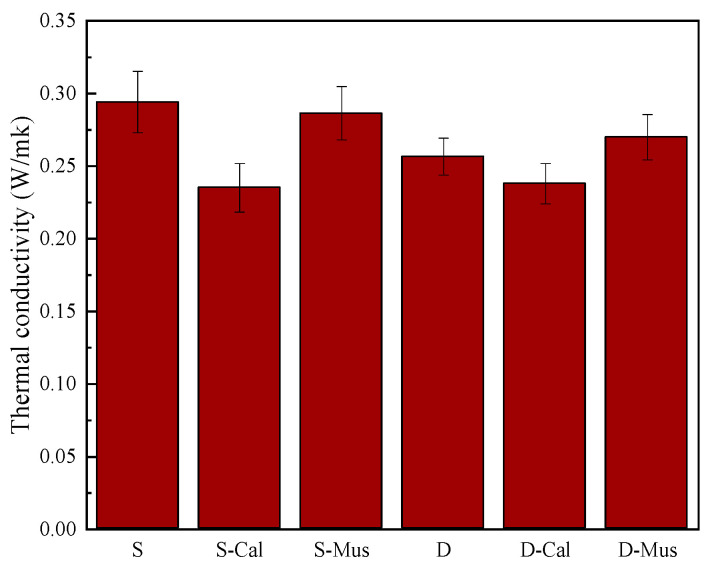
The effect of different mineral powders on the thermal conductivity of foam concrete.

The effect of different mineral powders on the thermal conductivity of foam concrete is shown in Figure 11. For the SDS group, the thermal conductivity of S was 0.2941 W/mk. S-Cal and S-Mus had 0.2352 W/mk and 0.2864 W/mk, which decreased by 20.03% and 17.41%, respectively. For the DTAB group, the thermal conductivity of D was 0.2566 W/mk. D-Cal and D-Mus had 0.2379 W/mk and 0.2699 W/mk. There was a decrease of 7.29% in D-Cal. The addition of mineral powders decreased the thermal conductivity of foam concrete. Thermal resistance as the reciprocal of thermal conductivity is proportional to the density of foam concrete, as lower density indicates greater porosity and hence lower thermal conductivity [36]. In addition, the influence of pore structure should also be noticed as the thermal conductivity may sometimes be increased by lower density due to the intensified pore connection [15,37]. The addition of mineral powder increases the number of pores in the foam concrete and reduces the thermal conductivity due to the very low thermal conductivity of air, and convection plays a greater role in the pores. In addition, the thermal conductivity of the DTAB group is less than the SDS group. The possible reason is that the porosity of the DTAB group is more than the SDS group.

### 3.5. Frost Resistance

**Figure 12:**
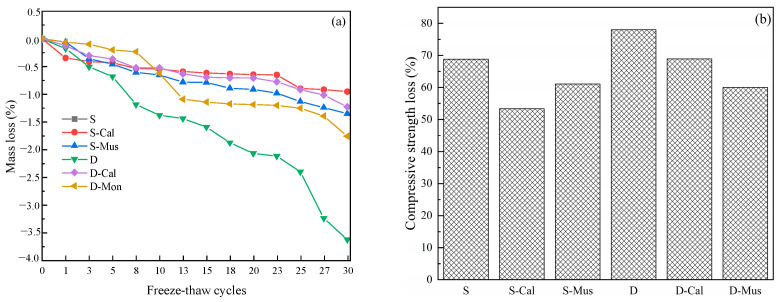
The effect of different mineral powders on the (**a**) mass loss of foam concrete in freeze–thaw cycles and (**b**) compressive strength loss after 30 cycles.

The effect of different mineral powders on the frost resistance of foam concrete is shown in Figure 12. It was found that the mass loss of all specimens increased with the number of freeze–thaw cycles. From Figure 12a, for the SDS group, the mass loss of S after 30 cycles was 1.90%. S-Cal and S-Mus were 0.96% and 1.35%, which decreased by 49.47% and 28.95%, respectively. For the DTAB group, the mass loss of D after 30 cycles was 3.63%. D-Cal and D-Mus had 1.23% and 1.79%, which decreased by 66.12% and 50.69%, respectively. As shown in Figure 12b, for the SDS group, the compressive strength loss of S after 30 cycles was 68.85%. S-Cal and S-Mus had 53.38% and 61.12%, which decreased by 22.47% and 11.23%, respectively. For the DTAB group, the compressive strength loss of D after 30 cycles was 78%. D-Cal and D-Mus had 68.95% and 59.97%, which decreased by 11.60% and 23.12%, respectively. There is a strong correlation between the frost resistance of foam concrete and water absorption [38,39]. Sun et al. [40] reached similar conclusions by studying the effect of different types of blowing agents on foam concrete. The addition of mineral powders reduced water absorption and therefore increased the frost resistance of foam concrete. In addition, the frost resistance of the SDS group was greater than that of the DTAB group because the water absorption of SDS was lower than that of the DTAB group and the strength was higher than that of the DTAB group.

### 3.6. Mechanisms of Mineral Powder Enhancement

#### 3.6.1. XRD Analysis Results

**Figure 13:**
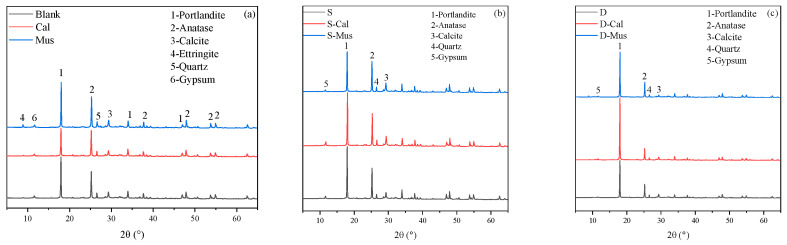
XRD spectra of (**a**) cement paste without foam, (**b**) SDS group and (**c**) DTAB group.

The XRD spectra of cement paste without foam and foam concrete of SDS and DTAB group for 28 days were tested and are shown in Figure 13. It was found that the main hydration products were calcium hydroxide, ettringite and gypsum. Furthermore, carbonation during the curing period resulted in the formation of calcium carbonate. It was noticed that no new products appeared in the specimens with 1% mineral powders with or without foaming agents.

#### 3.6.2. LF-NMR Analysis Results

**Figure 14:**
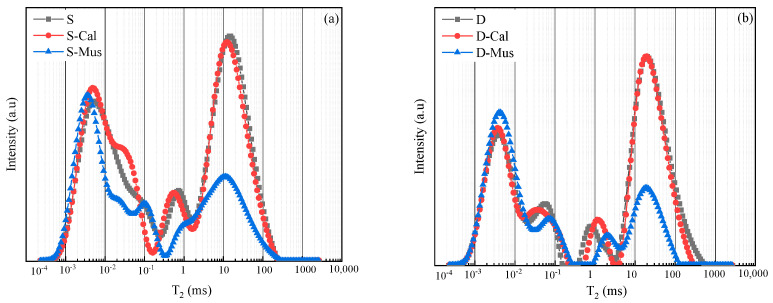
LF-NMR results of (**a**) SDS group and (**b**) DTAB group.

The LF-NMR technique was used to qualitatively characterize the pore size distribution in the SDS and DTAB groups and the results are shown in Figure 14. Gel water, capillary water and surface water corresponded to *T*_2_ values at approximately 0.01–1, 1–100 and 100–1000 ms, respectively [41]. By measuring the *T*_2_ relaxation times of water in saturated specimens, it was found that two groups of specimens exhibited *T*_2_ relaxation times in the range of 0.01–1 ms, the relaxation intensity of which was not changed when mineral powders were incorporated, indicating that the addition of mineral powders does not affect early-stage hydration. As revealed by the particle size test results, the particle size of muscovite was smaller than the cement particles, and thus the filling effect of Mus leads to a decrease in the number of capillary pores. Consequently, the *T*_2_ relaxation intensity of S-Mus and D-Mus is lower than that of S and D within the range of 100–1000 ms, respectively.

#### 3.6.3. FTIR Analysis Results

**Figure 15:**
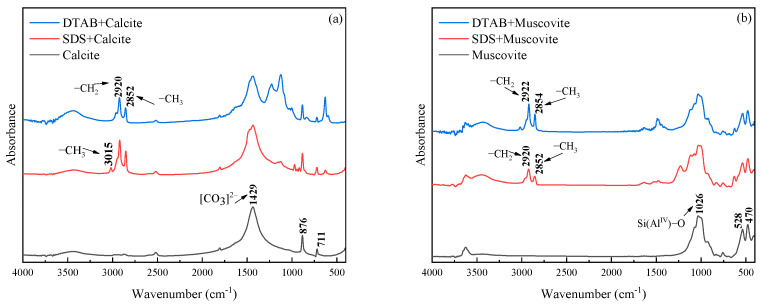
FTIR spectrum of (**a**) calcite and (**b**) muscovite.

The FTIR results after the interaction of anionic and cationic surfactants with mineral powders, as well as for the original calcite and muscovite, are shown in Figure 15, where no significant shifts in peak wavenumbers can be observed. From Figure 15a, for bare calcite, the bands at 876 cm^−1^ and 711 cm^−1^ are attributed to the deformation vibration of C-O in calcite and the bands at 1429 cm^−1^ correspond to the stretching vibration of C-O in calcite. After being treated with SDS, new bands emerged at 2920 cm^−1^ and 2852 cm^−1^, caused by the stretching vibration and asymmetric stretching vibration of the methyl group (CH_2_) in the SDS. These new bands indicate that SDS has been adsorbed on the surface of calcite. For DTAB, new bands emerge at 3015 cm^−1^, also demonstrating that the adsorption of DTAB occurs on the calcite surface.

From Figure 15b, there exist three main frequencies that reflect the crystal structure of muscovite: frequency at 748 cm^−1^ due to the vibration of the Al-O bond, frequency at 1026 cm^−1^ due to the vibration of the Si-O bond and frequency at 3624 cm^−1^ due to the vibration of −OH. With the treatment of SDS or DTAB, the characteristic bands of SDS or DTAB are at 2920 cm^−1^, 2852 cm^−1^ and 3015 cm^−1^. The FTIR spectra analysis result indicates that SDS and DTAB can be adsorbed on the surface of the calcite and muscovite.

#### 3.6.4. SEM Analysis Results

Overall, the addition of mineral powders did not affect the matrix of the foam concrete, either as a result of strength, hydration products or microscopic pores. The FTIR result indicates that mineral powders interact with cationic and anionic surfactants through physical adsorption. The possible mechanism of the effect of mineral powder on the properties of foam concrete is the interaction between mineral powder and foam through electrostatic adsorption.

**Figure 16:**
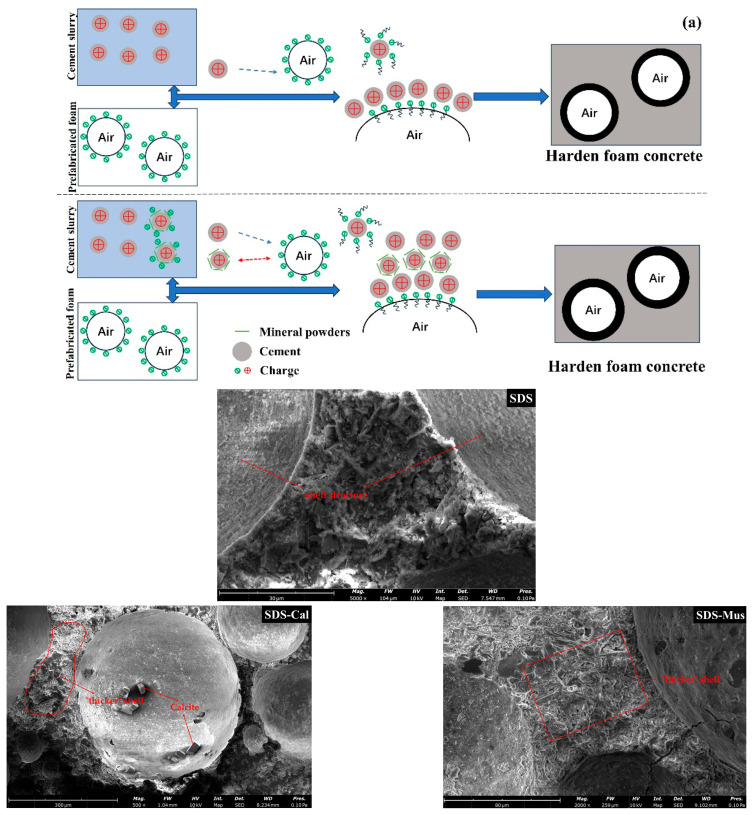

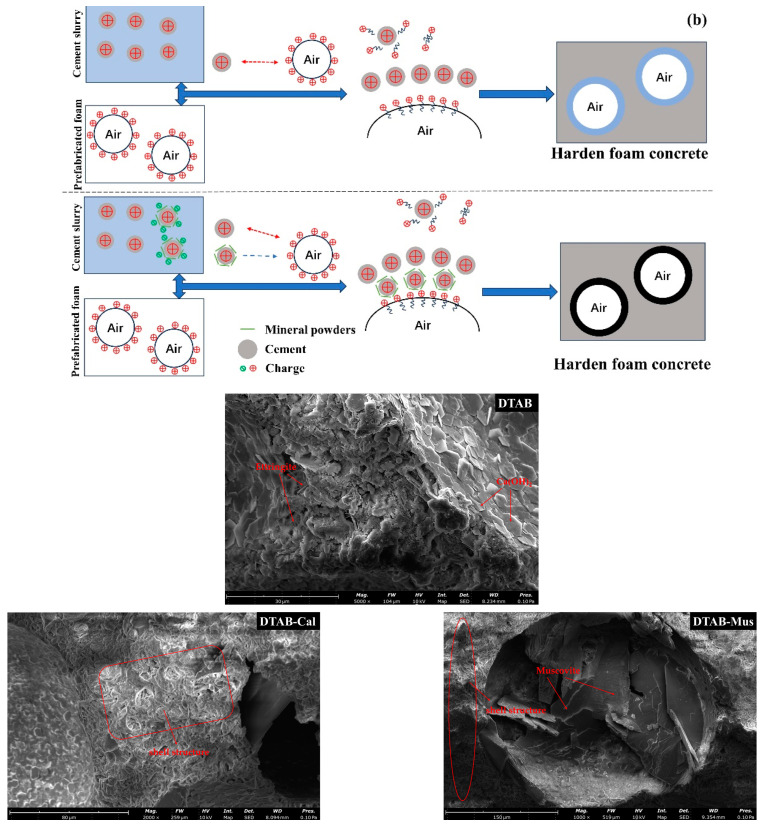
Schematic representation and SEM images of the formation of products on the pore walls of foam concrete prepared using mineral powders with anionic surfactant (**a**) and cationic surfactant (**b**).

The schematic formation and SEM images of products on the wall of air pores in foam concrete prepared using mineral powders with anionic surfactants such as SDS are shown in Figure 16a, referring to the mechanism diagram in the article by Xun [42] and Hou [17]. The cement particles had the opposite charge as the foaming agent, so cement particles were easily absorbed into the bubbles when cement paste was mixed with prefabricated foams. A shell structure surrounding the air pores was formed in the hardened foam concrete, and it would be separated from the cement matrix for a certain distance. Therefore, there was less ettringite but some C-S-H gels and calcium hydroxide at the interface between the cement matrix and air. Calcite and muscovite have negative Zeta sites at pH = 12.5, and thus there is an interaction with the positively charged portion of the cement surface through electrostatic adsorption. So, when cement and mineral powder are mixed, the mineral powder is easily adsorbed on the cement particles. When the cement paste with mineral powder is mixed with the foam prepared by SDS, a part of the cement particles adsorbed with mineral particles can be adsorbed on the cement particles of the bubble wall. So, a “thicker” shell is formed in the hardened foam concrete pore wall.

DTAB as the foaming agent is shown in Figure 16b. The cement particles had the same charge as the foaming agent, so cement particles were hardly absorbed onto the bubbles when cement slurry was mixed with prefabricated foams. So, there was a water−rich area near the pores in the fresh foam concrete. SO_4_^2−^ and AlO_2_^−^ were easy to migrate to the membrane nearby due to the positive charge of DTAB, which would be beneficial for the formation and enrichment of ettringite in the loose air pore walls. When the cement paste with mineral powder was mixed with the foam prepared by DTAB, due to electrostatic interactions, a part of the cement particles adsorbed with mineral particles could be adsorbed on the bubble wall. So, a shell is formed in the hardened foam concrete pore wall.

SEM images can illustrate the above mechanism and also how mineral powders were found on the hardened pore walls of the foam concrete.

## 4. Conclusions

In this study, the effect of mineral powders on the properties of foam concrete prepared with cationic and anionic surfactants was examined. The fluidity of the paste, mechanical properties, water absorption, pore characteristics, thermal conductivity and frost resistance were investigated. Meanwhile, the mechanism of a small amount of mineral powder to enhance the performance of foam concrete was proposed by analyzing the results of XRD, LF-NMR, FTIR and SEM. Based on the results of this study, the following conclusions can be drawn.

1. The addition of 1% calcite and muscovite does not affect the compressive strength of cement paste and does not produce new hydration products. Muscovite has water absorption capacity and reduces the fluidity of cement paste.
2. When SDS is used as a foaming agent to prepare foam concrete:
  The addition of 1% calcite increased the fluidity of fresh foam concrete by 10.29%. The dry density increased by 8.44%, the compressive strength/dry density ratio did not change much and water absorption decreased by 25.87%. The thermal conductivity decreased by 20.03% and the average size of the pores decreased by 16.43%. After 30 freeze–thaw cycles, the mass loss decreased by 49.47% and the strength loss decreased by 22.47%.
  The addition of 1% white muscovite decreased the flow of fresh foam concrete by 11.27%. Dry density increased by 6.86%, compressive strength/dry density ratio increased by 20.94% and water absorption decreased by 34.40%. The thermal conductivity was reduced by 17.41% and the average diameter of the pores was reduced by 10.75%. After 30 freeze–thaw cycles, the mass loss was reduced by 28.95% and the strength loss was reduced by 11.23%.
3. When DTAB is used as a foaming agent to prepare foam concrete:
  The addition of 1% calcite increased the fluidity of fresh foam concrete by 10.78%. The dry density increased by 6.86%, the compressive strength/dry density ratio increased by 27.20% and water absorption did not change much. The thermal conductivity decreased by 7.29% and the average size of the pores decreased by 9.51%. After 30 freeze–thaw cycles, the mass loss decreased by 16.12% and the strength loss decreased by 11.60%.
  The addition of 1% white muscovite decreased the flow of fresh foam concrete by 8.82%. Dry density increased by 59.31%, the compressive strength/dry density ratio increased by 29.12% and water absorption decreased by 28.83%. The thermal conductivity did not change much and the average diameter of the pores was reduced by 25.06%. After 30 freeze–thaw cycles, the mass loss was reduced by 11.60% and the strength loss was reduced by 23.12%.
4. In the same design dry density, foam concrete prepared with DTAB as foaming agent has higher porosity and larger pore size compared to SDS, which is the main reason for the difference in their performance.
5. The specific physical adsorption of calcite and muscovite with anionic or cationic surfactants also exists in the simulated environment of the cement pore solution. The addition of mineral powders promotes the formation of a shell structure around the foam due to electrostatic interactions and the capillary pore content in the foam concrete was reduced. Thus, the properties of foam concrete were enhanced, whether the foaming agent is an anionic or cationic surfactant.
  The mechanism of mineral-powder-reinforced foam concrete is complex, especially in the study of mineral particles to adsorb directionally on foam walls prepared with ionic surfactants in cementitious materials. More work is needed in the future.

## Figures and Tables

**Table 1:** Basic properties of cement.

Loss-on-Ignition (%)	SO_3_ (%)	MgO (%)	Specific Surface (m^2^/kg)	Initial Setting Time (min)	Final Setting Time (min)	Invariability	Chloride Ion Content (%)
3.54	1.85	0.76	337	197	247	Pass	0.015

**Table 2:** Compositions of foam concrete with different mineral powders.

Mixture	Cement (kg)	Mineral Powders (kg)	Water (kg)	W/C	Foam (m^3^)	Foaming Agent Amounts (L)
S	666.7	0	333	0.5	0.72	27.29
S-Cal	660	6.7				
S-Mus						
D	666.7	0				16.17
D-Cal	660	6.7				
D-Mus						

**Table 3:** The pore parameter of the specimens with the SDS group.

Pore Parameter	Specimens	Size		
		<500 μm	<1000 μm	Full
Number	S	3335	4190	4668
	S-Cal	4942	6102	6601
	S-Mus	4308	5174	5636
Frequency (%)	S	71.4	89.8	100
	S-Cal	74.9	92.4	100
	S-Mus	76.4	91.8	100
Porosity (%)	S	11.76	21.08	35.93
	S-Cal	17.98	30.50	42.46
	S-Mus	14.93	24.32	38.71
Average size (μm)	S	0.226	0.322	0.493
	S-Cal	0.233	0.320	0.412
	S-Mus	0.222	0.301	0.440

**Table 4:** The pore parameter of the specimens with the DTAB group.

Pore Parameter	Specimens	Size		
		<500 μm	<1000 μm	Full
Number	D	1444	1939	2770
	D-Cal	1672	2392	3477
	D-Mus	1840	2638	3692
Frequency (%)	D	52.1	70.0	100
	D-Cal	48.1	68.8	100
	D-Mus	49.8	71.5	100
Porosity (%)	D	5.02	10.58	52.32
	D-Cal	6.28	14.24	59.43
	D-Mus	6.86	15.87	52.25
Average size (μm)	D	0.222	0.349	1.209
	D-Cal	0.241	0.381	1.094
	D-Mus	0.239	0.385	0.906

## Data Availability

The original contributions presented in the study are included in the article, further inquiries can be directed to the corresponding author.

## References

[1] Nambiar E.K.K., Ramamurthy K. (2007). Air–Void Characterisation of Foam Concrete. Cem. Concr. Res..

[2] Koehler S.A., Hilgenfeldt S., Weeks E.R., Stone H.A. (2004). Foam Drainage On the Microscale II. Imaging Flow through Single Plateau Borders. J. Colloid Interface Sci..

[3] Cilek E.C., Karaca S. (2015). Effect of Nanoparticles on Froth Stability and Bubble Size Distribution in Flotation. Int. J. Miner. Process..

[4] Dhasindrakrishna K., Pasupathy K., Ramakrishnan S., Sanjayan J. (2020). Effect of Yield Stress Development On the Foam–Stability of Aerated Geopolymer Concrete. Cem. Concr. Res..

[5] Dhasindrakrishna K., Pasupathy K., Ramakrishnan S., Sanjayan J. (2021). Progress, Current Thinking and Challenges in Geopolymer Foam Concrete Technology. Cem. Concr. Compos..

[6] Dhasindrakrishna K., Ramakrishnan S., Pasupathy K., Sanjayan J. (2021). Collapse of Fresh Foam Concrete: Mechanisms and Influencing Parameters. Cem. Concr. Compos..

[7] Jones M.R., Ozlutas K., Zheng L. (2016). Stability and Instability of Foamed Concrete. Mag. Concr. Res..

[8] Gonzenbach U.T., Studart A.R., Tervoort E., Gauckler L.J. (2006). Ultrastable Particle–Stabilized Foams. Angew. Chem.–Int. Edit..

[9] Li X., Xiong Y., Chen D., Zou C. (2015). Utilization of Nanoparticle–Stabilized Foam for Gas Well Deliquification. Colloids Surf. A Physicochem. Eng. Asp..

[10] Murray B.S., Ettelaie R. (2004). Foam Stability: Proteins and Nanoparticles. Curr. Opin. Colloid Interface Sci..

[11] Chen S., Liu H., Yang J., Zhou Y., Zhang J. (2019). Bulk Foam Stability and Rheological Behavior of Aqueous Foams Prepared by Clay Particles and Alpha Olefin Sulfonate. J. Mol. Liq..

[12] Zhu J., Yang Z., Li X., Hou L., Xie S. (2019). Experimental Study On the Microscopic Characteristics of Foams Stabilized by Viscoelastic Surfactant and Nanoparticles. Colloids Surf. A Physicochem. Eng. Asp..

[13] Binks B.P., Lumsdon S.O. (2000). Influence of Particle Wettability on the Type and Stability of Surfactant−Free Emulsions. Langmuir.

[14] Dandamudi C., Beniah G., Lee J., Lyon B., Han J.J., Pennell K., Lynd N., Johnston K. (2018). Colloidal Stabilization of Silica and Iron Oxide Nanoparticles in Highly Concentrated Divalent Salts. Abstracts of Papers of the American Chemical Society.

[15] Hajimohammadi A., Ngo T., Mendis P., Kashani A., van Deventer J.S.J. (2017). Alkali Activated Slag Foams: The Effect of the Alkali Reaction On Foam Characteristics. J. Clean. Prod..

[16] Horozov T.S. (2008). Foams and Foam Films Stabilised by Solid Particles. Curr. Opin. Colloid Interface Sci..

[17] Hou L., Li J., Lu Z., Niu Y. (2021). Influence of Foaming Agent On Cement and Foam Concrete. Constr. Build. Mater..

[18] Wei S., Yi D., Changwen M., Jiaping L., Guotang Z., Jinyang J., Yunsheng Z. (2018). Application of Organic- and Nanoparticle-Modified Foams in Foamed Concrete: Reinforcement and Stabilization Mechanisms. Cem. Concr. Res..

[19] Guo S., Wang W., Jia Z., Qi X., Zhu H., Liu X. (2023). Nanoparticle−Stabilized Foam with Controllable Structure for Enhanced Foamed Concrete. Constr. Build. Mater..

[20] Li G., Tan H., He X., Zhang J., Deng X., Zheng Z. (2022). Research On the Properties of Wet−Ground Waste Limestone Powder as Foam Stabilizer in Foamed Concrete. Constr. Build. Mater..

[21] Krämer C., Kowald T.L., Trettin R.H.F. (2015). Pozzolanic Hardened Three−Phase−Foams. Cem. Concr. Compos..

[22] Krämer C., Schauerte M., Kowald T.L., Trettin R.H.F. (2015). Three−Phase−Foams for Foam Concrete Application. Mater. Charact..

[23] Krämer C., Schauerte M., Müller T., Gebhard S., Trettin R. (2017). Application of Reinforced Three−Phase−Foams in Uhpc Foam Concrete. Constr. Build. Mater..

[24] (2013). Foaming Agents for Foamed Concrete.

[25] Amran Y.H.M., Farzadnia N., Abang Ali A.A. (2015). Properties and Applications of Foamed Concrete; A Review. Constr. Build. Mater..

[26] Kearsley E.P., Wainwright P.J. (2001). The Effect of High Fly Ash Content On the Compressive Strength of Foamed Concrete. Cem. Concr. Res..

[27] (2014). Technical Specification for Application of Foamed Concrete.

[28] Gu X., Wang S., Liu J., Wang H., Xu X., Wang Q., Zhu Z. (2023). Effect of Hydroxypropyl Methyl Cellulose (Hpmc) as Foam Stabilizer On the Workability and Pore Structure of Iron Tailings Sand Autoclaved Aerated Concrete. Constr. Build. Mater..

[29] (2008). Thermal Insulation Determination of Steady State Thermal Resistance and Related Properties Guarded Hot Plate Apparatus.

[30] Ouyang X., Koleva D.A., Ye G., van Breugel K. (2017). Understanding the Adhesion Mechanisms Between C S H and Fillers. Cem. Concr. Res..

[31] Guo R., Xue C., Guo W., Wang S., Shi Y., Qiu Y., Zhao Q. (2023). Preparation of Foam Concrete From Solid Wastes: Physical Properties and Foam Stability. Constr. Build. Mater..

[32] Lei L., Plank J. (2014). A Study On the Impact of Different Clay Minerals On the Dispersing Force of Conventional and Modified Vinyl Ether Based Polycarboxylate Superplasticizers. Cem. Concr. Res..

[33] Kearsley E.P., Wainwright P.J. (2002). The Effect of Porosity On the Strength of Foamed Concrete. Cem. Concr. Res..

[34] Kolias S., Georgiou C. (2005). The Effect of Paste Volume and of Water Content On the Strength and Water Absorption of Concrete. Cem. Concr. Compos..

[35] Song N., Li Z., Yi W., Wang S. (2022). Properties of Foam Concrete with Hydrophobic Starch Nanoparticles as Foam Stabilizer. J. Build. Eng..

[36] Asadi I., Shafigh P., Abu Hassan Z.F.B., Mahyuddin N.B. (2018). Thermal Conductivity of Concrete—A Review. J. Build. Eng..

[37] Sang G., Zhu Y., Yang G., Zhang H. (2015). Preparation and Characterization of High Porosity Cement−Based Foam Material. Constr. Build. Mater..

[38] Nambiar E.K.K., Ramamurthy K. (2007). Sorption Characteristics of Foam Concrete. Cem. Concr. Res..

[39] Tikalsky P.J., Pospisil J., MacDonald W. (2004). A Method for Assessment of the Freeze–Thaw Resistance of Preformed Foam Cellular Concrete. Cem. Concr. Res..

[40] Chao S., Yu Z., Jian G., Yamei Z., Guoxing S. (2018). Effects of Foaming Agent Type On the Workability, Drying Shrinkage, Frost Resistance and Pore Distribution of Foamed Concrete. Constr. Build. Mater..

[41] Yao Y., Liu D., Che Y., Tang D., Tang S., Huang W. (2010). Petrophysical Characterization of Coals by Low−Field Nuclear Magnetic Resonance (NMR). Fuel.

[42] Zhong X., Liu D., Shi X., Zhao H., Pei C., Zhu T., Shao M., Zhang F. (2018). Characteristics and Functional Mechanisms of Clay−Cement Stabilized Three−Phase Nitrogen Foam for Heavy Oil Reservoir. J. Pet. Sci. Eng..

